# Presequence translocase-associated motor subunits of the mitochondrial protein import apparatus are dual-targeted to mitochondria and plastids

**DOI:** 10.3389/fpls.2022.981552

**Published:** 2022-11-11

**Authors:** Mabel Gill-Hille, Andre Wang, Monika W. Murcha

**Affiliations:** ^1^ School of Molecular Sciences, The University of Western Australia, Perth, WA, Australia; ^2^ Australian Research Council (ARC) Centre of Excellence in Plant Energy Biology, The University of Western Australia, Perth, WA, Australia

**Keywords:** mitochondria, plastids, dual-targeting, PAM, protein import apparatus

## Abstract

The import and assembly of most of the mitochondrial proteome is regulated by protein translocases located within the mitochondrial membranes. The Presequence Translocase-Associated Motor (PAM) complex powers the translocation of proteins across the inner membrane and consists of Hsp70, the J-domain containing co-chaperones, Pam16 and Pam18, and their associated proteins Tim15 and Mge1. In Arabidopsis, multiple orthologues of Pam16, Pam18, Tim15 and Mge1 have been identified and a mitochondrial localization has been confirmed for most. As the localization of Pam18-1 has yet to be determined and a plastid localization has been observed for homologues of Tim15 and Mge1, we carried out a comprehensive targeting analysis of all PAM complex orthologues using multiple *in vitro* and *in vivo* methods. We found that, Pam16 was exclusively targeted to the mitochondria, but Pam18 orthologues could be targeted to both the mitochondria and plastids, as observed for the PAM complex interacting partner proteins Tim15 and Mge1.

## Introduction

**Figure 1:**
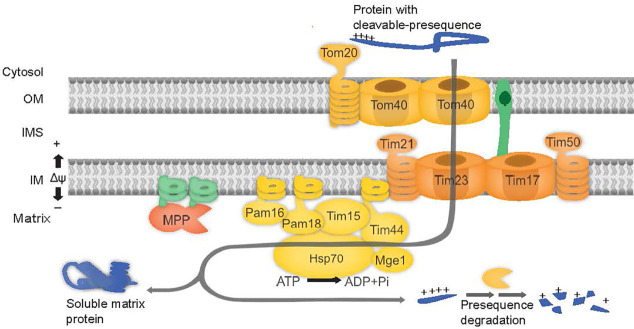
PAM complex subunits are involved in mitochondrial protein import. The preprotein binds to the outer membrane (OM) at the TOM complex and is translocated across Tom40. The preprotein is directed to the TIM17:23 complex, consisting of proteins Tim23, Tim17, Tim21, and Tim50. Release of proteins into matrix is powered by ATP hydrolysis *via* Hsp70 in the PAM complex consisting of proteins Tim44, Tim15, Pam16 and Pam18, Mge1 and Hsp70.

Mitochondria and plastids evolved from two independent endosymbiotic events, approximately 2 and 1 billion years ago respectively (McFadden, 1999). Over the course of evolution, most of their genomes was lost, requiring organelle-specific protein import components and mechanisms to function. Protein import involves a multitude of chaperones, receptors, transporters and proteases that deliver nuclear encoded cytosolic proteins to the organelle, translocate them through membranes, process and sort the protein to its final intra-organellar destination (Ghifari et al., 2018). For most of the mitochondrial proteome, an N-terminal targeting signal delivers the precursor protein to the mitochondria, allowing it to translocate across the Translocase of the Outer Membrane (TOM) complex pore, further directing it to the TIM17:23 inner membrane complex. This complex consists of the inner membrane spanning channel proteins Tim17 and Tim23, and associated proteins such as Tim21 and Tim50 that stabilize the complex and the Presequence Assisted Motor (PAM) complex that powers the translocation of preproteins through the inner membrane channel (Neupert and Herrmann, 2007). In yeast, the PAM complex consists of mtHsp70, Tim44, Tim15, Pam18, Pam16, Pam17, and Mge1. MtHsp70 is the central motor that interacts with the precursor protein passing through TIM17:23 channel and powers translocation of proteins into the matrix by ATP hydrolysis requiring co-chaperones and nucleotide exchange factors (Kampinga and Craig, 2010). The co-chaperone Pam18, stimulates the ATPase activity of mtHsp70 (Truscott et al., 2003), whilst Pam16 tethers Pam18 to the PAM complex (Pais et al., 2011) and Pam17 stabilizes the complex (Sanjuán Szklarz et al., 2005; van der Laan et al., 2005). Mge1, catalyzes the exchange of ADP for ATP allowing release and reload of the substrate (Liu et al., 2003) (**Figure 1**). Tim44 acts as a scaffold protein to recruit mtHsp70 (De Los Rios et al., 2006; Schiller et al., 2008) whilst Tim15, (also named Zim17, ZR, HEP1) is thought to stabilize this complex (Sanjuán Szklarz et al., 2005; van der Laan et al., 2005).

Pam18 and Pam16 belong to the DnaJ and DnaJ-like family of proteins respectively (Hennessy et al., 2005). J-domain proteins contain a canonical tripeptide HPD motif of Histidine (H), Proline (P), and Aspartate (D) residues essential for stimulating the ATPase activity of Hsp70 (Truscott et al., 2003; Hennessy et al., 2005). Pam16, lacking the HPD motif forms a heterodimer with Pam18 and in doing so, stimulates the activity of Pam18 (Liu et al., 2004). Both Pam16 and Pam18 areessential for yeast viability (Frazier et al., 2003; Truscott et al., 2003) and are anchored to the inner membrane *via* an N-terminal transmembrane domain (Mokranjac et al., 2006) (**Figure 1**).

The *Arabidopsis thaliana* (Arabidopsis) genome encodes for three putative homologues of Pam18 (Pam18-1, Pam18-2 and Pam18-3) and two of Pam16 (Pam16-1 and Pam16-2), whilst no Pam17 orthologue could be identified by sequence homology (Murcha et al., 2015). Pam16-1 was initially characterized by a forward genetic screen mutant, named *muse5*-1, which displays delayed growth and an enhanced stress response (Huang et al., 2013). A T-DNA insertion line for Pam16-2 was also characterized, which did not display any observable phenotype, and the double knock-out line *muse5-1*::*pam16-2* was embryonically lethal suggesting functional overlap (Huang et al., 2013). Localization using biolistic transformation of GFP tagged Pam16-1 and Pam16-2 into onion epidermal cells identified both proteins to be targeted to mitochondria (Huang et al., 2013; Tamadaddi et al., 2021). GFP tagged constructs of Pam18-2 and Pam18-2 also identified both proteins to be targeted to the mitochondria in root cells of stable GFP fusion lines, whilst no GFP fluorescence targeting could be observed for Pam18-1 (Tamadaddi et al., 2021). Three genes encode for Tim15, Tim15-2 (also known as ZR3) shown to be localized to the mitochondria, is able to functionally complement a Tim15 yeast deletion strain (Kluth et al., 2012). Tim15-1/ZR1 and Tim15-3/ZR2 were shown to targeted to the chloroplasts (Kluth et al., 2012). In plants, two homologues exist for Mge1, Mge1 and Mge2 both shown to be mitochondrial proteins by GFP-tagged localization assays and proteomic analysis (Hu et al., 2012). Interestingly, Mge1 was shown to be dual-targeted to mitochondria and plastids in Arabidopsis cell suspensions and by protein uptake assays into isolated organelles (Van Aken et al., 2009; Langner et al., 2014). The undetermined localization of Pam18-1, the dual-targeting of Mge1 and the plastid localization of Tim15-1/3 proposes that PAM subunits may play a role in plastids. Here, we reexamined the targeting ability of all PAM complex homologues, Pam16-1, Pam16-2, Pam18-1, Pam18-2, Pam18-3, Tim15-1, Tim15-2, Tim15-3, Mge1 and Mge2 using GFP-tagged biolistic transformation stable transgenic GFP fusion lines and *in vitro* protein uptake assays into isolated organelles. We confirmed that Pam16-1 and Pam16-2 were exclusively targeted to the mitochondria, whilst Pam18-1, Pam18-2 and Pam18-3 could be dual-targeted to both the mitochondria and plastid, depending on the methodology used and tissue types investigated which suggests a possible role of the Pam18 co-chaperones in plastids.

## Materials and methods

### Bioinformatic analysis

The sequences for all Arabidopsis orthologues were obtained from TAIR (https://www.arabidopsis.org/). Homologues from *Homo sapiens, Caenorhabditis elegans, Cyanobacteria, Oryza sativa, Zea mays*, and *Selaginella moellendorffii* were obtained by BLAST in NCBI using standard parameters (https://www.ncbi.nlm.nih.gov/) (**Supplementary Table 1**). Protein sequences were aligned using ClustalW in MEGA7 (Kumar et al., 2016). Phylogenetic trees were constructed using the Maximum likelihood method based on the Jones-Taylor-Thornton (JTT) model and bootstrap consensus was inferred from 1000 replicates. Domains and motifs were analysed using InterPro (https://www.ebi.ac.uk/interpro/) and ScanProsite (https://prosite.expasy.org/scanprosite/) (**Supplementary Table 1**). Tertiary structures were predicted using Phyre2 (Kelley et al., 2015) and overlapped using Maestro (www.schrodinger.com).

### Cloning and constructing of vectors

All clones were amplified from Arabidopsis cDNA with or without a stop codon using primers listed in **Supplementary Table 2** and recombined into pDONR201 or pDONR207 using Gateway^®^ Technology (Invitrogen). For GFP-tagged protein localization, cDNA clones were recombined into pDEST-cGFP (Carrie et al., 2007), for protein translation into pDEST14, for stable GFP fusion lines pGWB5 (Nakagawa et al., 2009). For radiolabelled protein translation, Pam16-1, Pam16-2, Pam18-1, Pam18-2, and Tim15-1 required additional methionines for visualization which were introduced to the N-terminus by PCR orusing the Agilent QuickChange II XL Site Directed Mutagenesis Kit with primers listed in **Supplementary Table 2**. Mge1 and Cge2 cDNA clones were both obtained from ABRC.

### Plant material and growth conditions

Arabidopsis *cv* Columbia (Col-0) plants were grown on half strength Murashige & Skoog (0.5 X MS) media agar plates or water culture pots containing 1.5% (w/v) sucrose under long day (16 h day cycle) growth conditions, with a light intensity of 100 µE m^-2^ s^-1^ at 22°C.

### Biolistic transformation

GFP plasmids were biolistically transformed into 5-day old Arabidopsis PSBD wild-type cell culture (Menges and Murray, 2002) with either a mitochondrial RFP marker (AOX-RFP), or a plastid RFP marker (SSU-RFP) (Carrie et al., 2010) as described previously (Duncan et al., 2015). Biolistic transformation was carried out using the Bio-Rad Model PDS-1000/He Biolistic Particle Delivery System at a helium pressure of 1400 kPa, according to the manufacturer’s instructions. Transformed cells were placed in the dark at 26°C overnight prior to imaging. GFP and RFP fluorescence was visualized at 460-480 nm and 570-625 nm respectively using the BX61 Olympus confocal microscope. Images were captured using 100X magnification.

### Generation of stable GFP lines

Stable transgenic C-terminal GFP fusions were created by Agrobacterium floral dip as described previously (Clough and Bent, 1998). For confirming plastid localisation, GFP fusion lines were crossed to a SSU-RFP stable transgenic line (Carrie et al., 2007). Root, guard, and epidermal cells were imaged as above.

### Organelle isolation

Mitochondria were isolated from 14-day old Arabidopsis seedlings grown in 0.5 X MS liquid media as previously described (Duncan et al., 2015). Plastids were isolated from 14-day old seedlings grown on 0.5X MS plates as previously described (Aronsson and Jarvis, 2002)

### Protein uptake assays

[^35^S]-methionine radiolabelled proteins were translated using the TNT^®^ Coupled Reticulocyte system (Promega) according to manufacturer’s instructions. Protein uptake assays were carried out into either freshly isolated mitochondria or freshly isolated plastids as previously described (Duncan et al., 2015). To confirm the intra-mitochondrial localization of imported protein, the outer membrane was ruptured by osmotic shock following import and prior to the addition of Proteinase K (PK) as detailed previously (Duncan et al., 2015). Plastid protein uptake assays were carried out by incubating precursor protein with isolated plastids (30 ug) in 3 mM ATP, 10 mM Met, 10 mM Cys, 20 mM potassium gluconate, 10 mM NaHCO_3_, 3 mM MgSO_4_, 330 mM sorbitol, 50 mm HEPES-KOH, pH 8, 0.2% BSA, and 50 mM ascorbic acid) for 25°C for 5 min with gentle agitation. Plastids were and washed in cold 50 mM HEPES-KOH, pH 8, 0.3 m sorbitol, and 3 mm MgSO_2_ prior to treatment with thermolysin (10 μg) and CaCl_2_ (5 mm) for 15 min on ice. EDTA was added to stop the reaction and plastids pelleted. All import samples were resolved by SDS-PAGE (16% (w/v) Acrylamide SDS-PAGE gels or 16.5% (w/v) Acrylamide Tricine SDS-PAGE as described in (Schägger, 2006), dried, and exposed to a phosphor-imaging screen for 24 h.

## Results

### Phylogenetic analyses and protein characterization of the Arabidopsis PAM subunits

Pam16 and Pam18 of the PAM complex modulate mtHSP70 activity along with Tim15 and the nucleotide exchange factor, Mge1 required for the import of precursor proteins into the mitochondrial matrix to (**Figure 1**). The Arabidopsis genome encodes for two Pam16 (Pam16-1 and Pam16-2) and three Pam18 orthologues (Pam18-1, Pam18-2, and Pam18-3). Phylogenetic analysis indicates that Pam16 and Pam18 branch independently and are conserved across species (**Supplementary Figure 1A**). Arabidopsis encodes for three Tim15 orthologues (Tim15-1, Tim15-2 and Tim15-3), Tim15-2 (also known as ZR3) branches with human and yeast (*Saccharomyces cerevisiae*) Tim15 (also known as Hep1) whilst Tim15-1 and Tim15-3 branch distinctly to Tim15-2 and are conserved in the early land plant *Selaginella moellendorffii*, and monocots *Zea mays* and *Oryza sativa* (**Supplementary Figure 1B**) Pam16 and Pam18 orthologues contain the conserved intermembrane space (IMS) domain, J-domain, and a predicted N-terminal transmembrane region (**Supplementary Figure 1C**). Tim15 orthologues contain a zinc finger motif domain and a conserved C-C motif, present in human and yeast Tim15/HEP1 (**Supplementary Figure 2**). Structural predictions using Phyre2 (Kelley et al., 2015) show similarity for all Arabidopsis Pam16, Pam18, and Tim15 orthologues (green) when overlaid on their yeast counterpart (pink) (**Supplementary Figure 1D**).

### Pam16 orthologues are targeted to the mitochondria whilst Pam18 orthologues are dual-targeted to mitochondria and plastid

**Figure 2:**
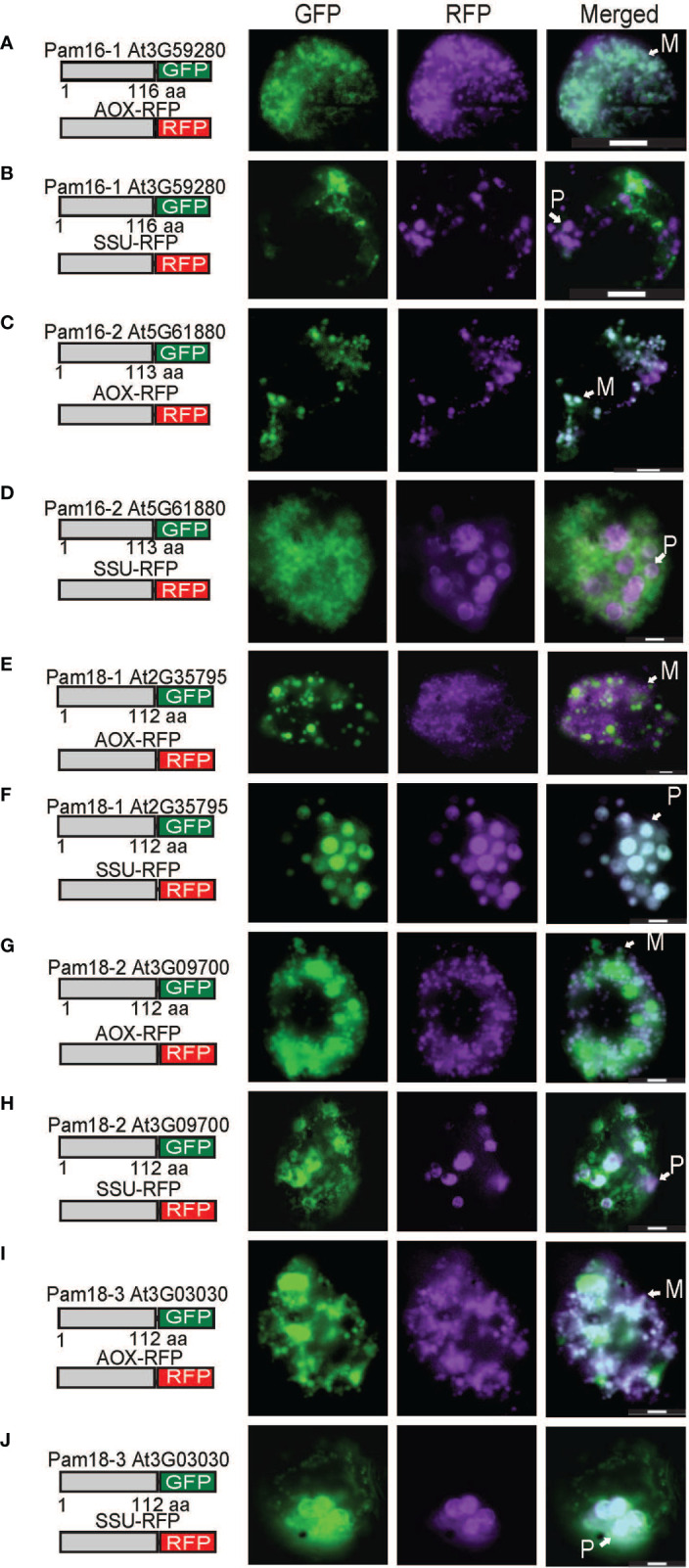
Biolistic transformation and localisation assays of Arabidopsis Pam16 and Pam18 GFP. C-terminal GFP fusions transformed into Arabidopsis cell suspensions alongside a mitochondrial targeted RFP (AOX-RFP) or plastid targeted RFP (SSU-RFP). Scale bar indicates 20 μm. M, mitochondria; P, plastid. Pam16-1 **(A, B)**. Pam16-2 **(C, D)**. Pam18-1 **(E, F)**. Pam18-2 **(G, H)**. Pam18-3 **(I, J)**.

The localization of C-terminal GFP-tagged Pam16 and Pam18 orthologues was examined using biolistic transformation of Arabidopsis cell suspensions. Full length GFP fusions were co-transformed with mitochondrial (AOX-RFP) or plastid (SSU-RFP) controls (**Figure 2**) (Carrie et al., 2010). The localization of Pam16-1 and Pam16-2 shows exclusive targeting to mitochondria as determined by co-localization with AOX-RFP (**Figures 2A–D**). Pam18-1-GFP fluorescence showed no overlay with AOX-RFP (**Figure 2E**) but did with SSU-RFP suggesting that Pam18-1 is targeted exclusively to the plastids (**Figure 2F**). Localization of Pam18-2 and Pam18-3 showed co-localization with both AOX-RFP and SSU-RFP suggesting dual-targeting to both mitochondria and plastid (**Figures 2G–J**).

### PAM interacting proteins Tim15 and Mge1 target to the mitochondria and plastids

**Figure 3:**
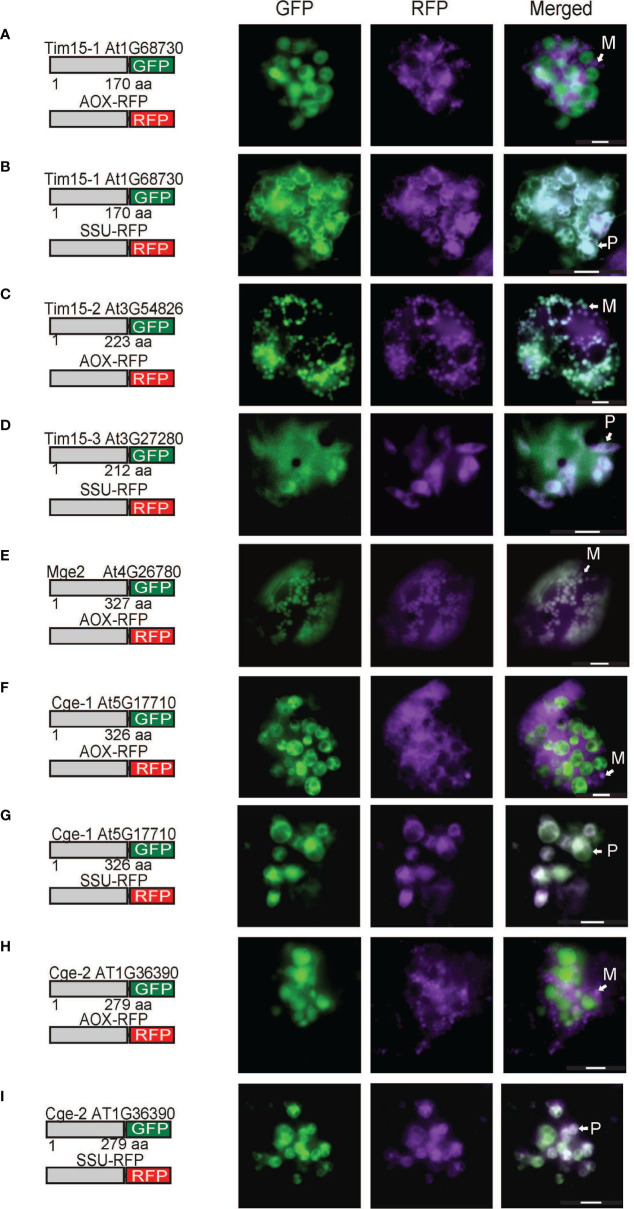
Biolistic transformation and localisation assays of Arabidopsis Tim15, Mge2 and Cge1/2 GFP. C-terminal GFP fusions transformed into Arabidopsis cell suspensions alongside a mitochondrial targeted RFP (AOX-RFP) or plastid targeted RFP (SSU-RFP). Tim15-1 **(A, B)**. Tim15-2 **(C)**. Tim15-3 **(D)**. Mge2 **(E)**. Cge1 **(F, G)**. Cge2 **(H, I)**. Scale bar indicates 20 μm. M, mitochondria; P, plastid.

As the plastid localization of Pam18 orthologues was unexpected, we tested the targeting of PAM Tim15, Mge2 and the plastid Mge1/2 counterparts, GrpE-like protein Cge1 and Cge2 (de Luna-Valdez et al., 2019). Tim15-1 exhibited exclusively plastid localization (**Figures 3A,B**), Tim15-2 co-localized to mitochondria (**Figure 3C**) and Tim15-3 co-localized to plastids and cytosol (**Figure 3D**). Multiple studies have comprehensively determined Mge1 to be dual-targeted to both mitochondria and plastids (Van Aken et al., 2009; Baudisch et al., 2014; Sharma et al., 2018) therefore we only tested Mge2 localization and determined it to target to the mitochondria (**Figure 3E**). Cge1 and Cge2 co-localized exclusively to the plastid (**Figures 3F–I**).

### Stable transgenic lines of Pam18 GFP show dual-targeting to the mitochondria and plastids

**Figure 4:**
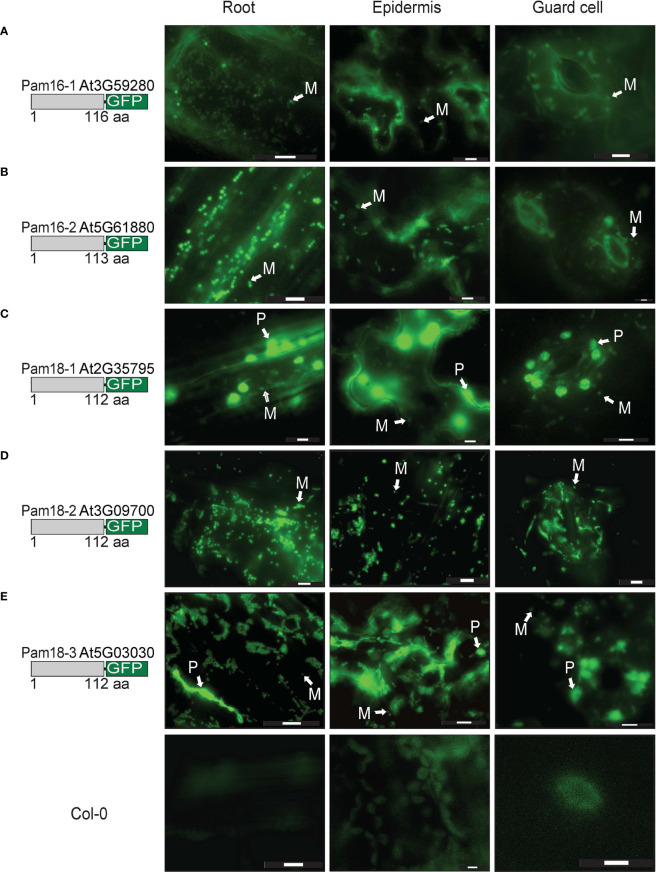
Pam16 and Pam18 GFP in stable transgenic Arabidopsis seedlings. Images taken from 3 weeks old Arabidopsis T2 transformants of root, epidermal, and guard cells Pam16-1 **(A)**. Pam16-2 **(B)**. Pam18-1 **(C)**. Pam18-2 **(D)**. Pam18-3 **(E)**. Scale bar indicates 20 μM. M, mitochondria, P, plastid.

Due to the unexpected dual-targeting of Pam18 using transient GFP fusions, we generated stable transgenic Arabidopsis lines for all Pam16 and Pam18 orthologues fused to GFP at the C-terminus (**Figure 4**; **Supplementary Figure 3**). Analysis of Pam16-1GFP and Pam16-2GFP stable lines indicated a typical mitochondrial-like localization of protein in root cells, epidermis, and guard cells (**Figures 4A, B**). Pam18-1GFP was observed to be targeted to both the mitochondria and larger plastid-like organelles in root, epidermis, and guard cells (**Figure 4C**). Pam18-2GFP was observed to be targeted exclusively to mitochondria in root, epidermis, and guard cells (**Figure 4D**). Pam18-3GFP was observed to be targeted to both the mitochondria and larger plastid-like organelles in root, epidermis, and guard cells (**Figure 4E**).

**Figure 5:**
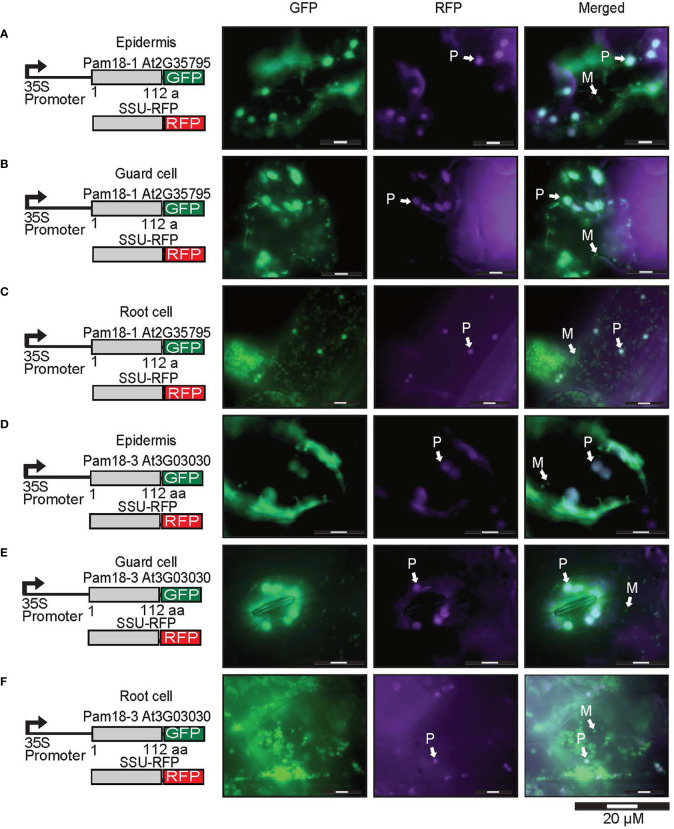
Pam18-1 and Pam18-3 GFP with SSU-RFP in stable transgenic Arabidopsis seedlings. Images taken from 3 weeks old Arabidopsis T2 transformants of root, epidermal, and guard cells Pam18-1 **(A–C)**. Pam18-3 **(D–F)**. Scale bar indicates 20 μM. M, mitochondria, P, plastid.

**Figure 6:**
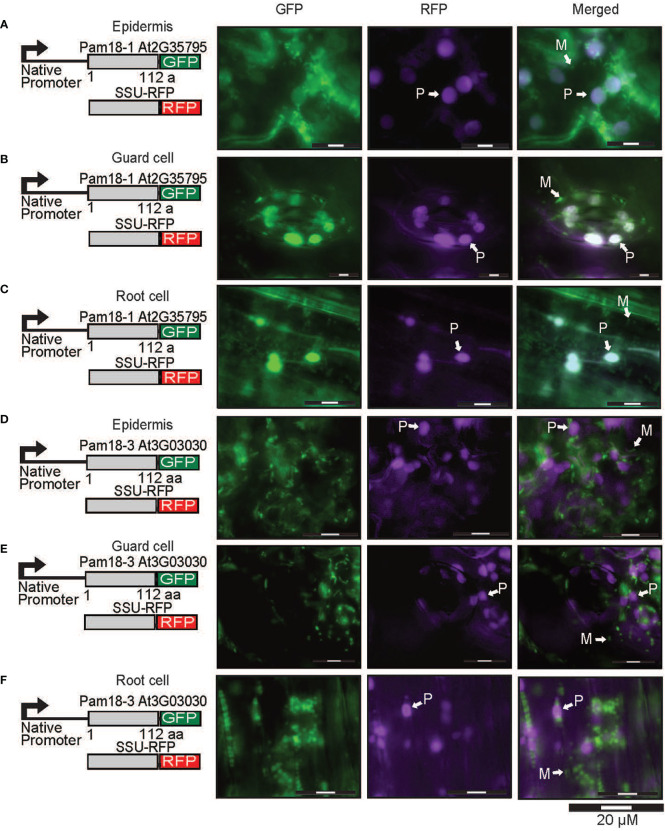
Pam18-1 and Pam18-3 GFP under endogenous promoters and plastid targeted-RFP (SSU-RFP) in stable transgenic Arabidopsis seedlings. Images taken from 3 weeks old Arabidopsis T2 transformants of root, epidermal, and guard cells Pam18-1 **(A–C)**. Pam18-3 **(D–F)**. Scale bar indicates 20 μM. M, mitochondria, P, plastid.

**Figure 7:**
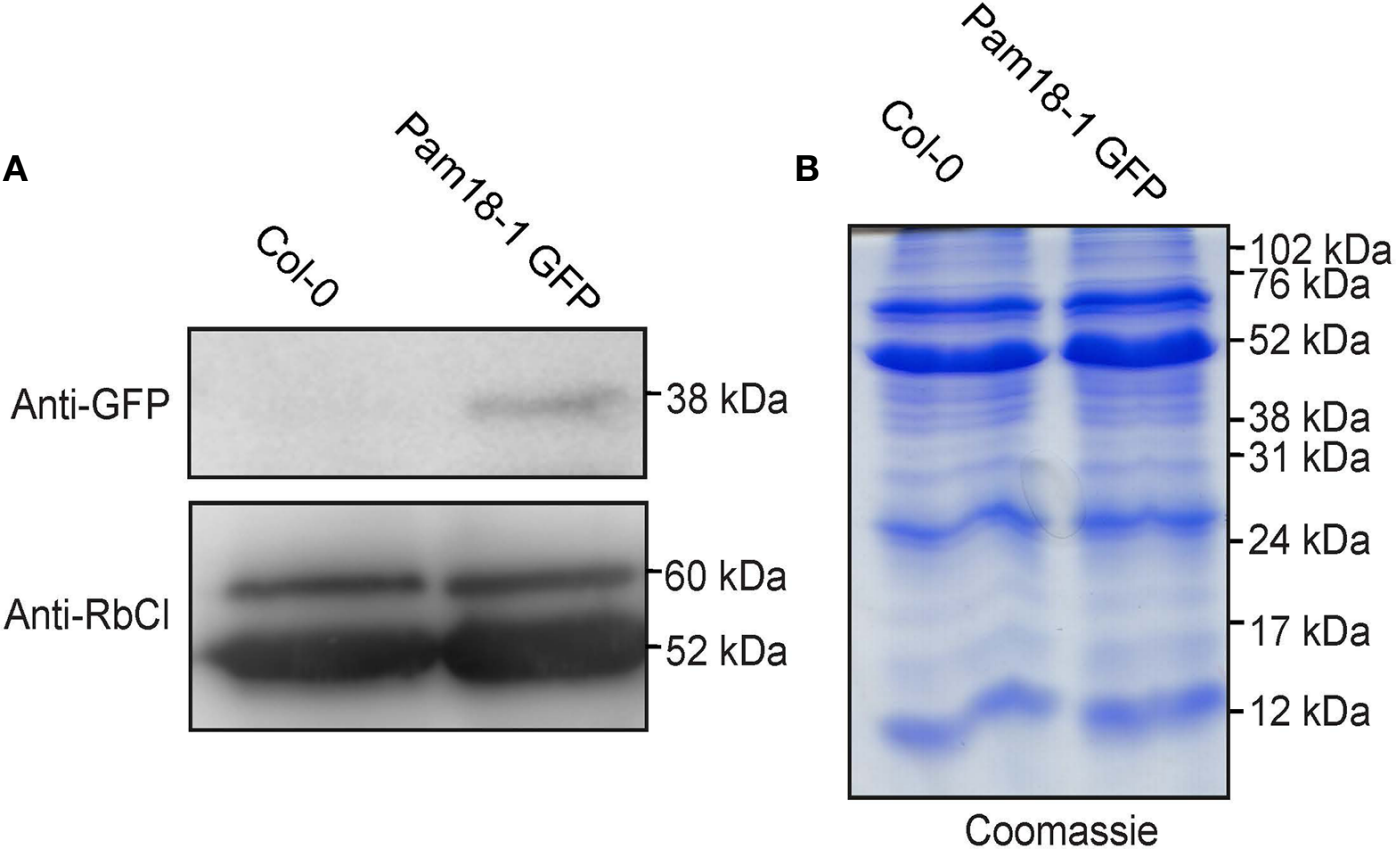
Immunodetection of Pam18-1 GFP under the expression of its endogenous promoter in isolated plastids. **(A)** Chloroplasts were isolated from 2 weeks old seedlings and immunodetected with anti-GFP and anti-RbCl. **(B)** Coomassie staining confirms equal protein loading and integrity.

To confirm that the larger organellar structures that we observed targeting to were plastids, Pam18-1 and Pam18-3 GFP lines were crossed with a stable SSU-RFP line (Carrie et al., 2010) (**Figure 5**, **Supplementary Figure 4**). Imaging of these double GFP/SSU-RFP tagged lines showed mitochondrial-like GFP localization patterns and a clear overlay GFP/RFP overlay in root, epidermal and guard cells (**Figures 5A–F**), confirming that Pam18-1 and Pam18-3 are dual-targeted to mitochondria and plastids as observed with transient transformations Pam18-1 and Pam18-3FGFP fusions were also tested under their native promoters and crossed with the same SSU-RFP line. Under a native promoter, Pam18-1, was found to be exclusively mitochondrial-located in epithelial cells (**Figure 6A**, **Supplementary Figure 4**) and dual-targeted in guard cells and root cells (**Figures 6B,C**). Under a native promoter, Pam18-3 was observed to be targeted to mitochondria in all cell types investigated (**Figures 6D–F**). To confirm that the localization was to plastids, immunodetection was carried out against isolated plastids from 14-day old Pam18-1 GFP seedlings. Immunodetection with anti-GFP indicated a band ~38 kDa (**Figure 7A**) whilst immunodetection using RbCL, and Coomassie staining confirmed equally loading (**Figures 7A, B**).

### Pam18 orthologues can be imported into isolated mitochondria and plastids

**Figure 8:**
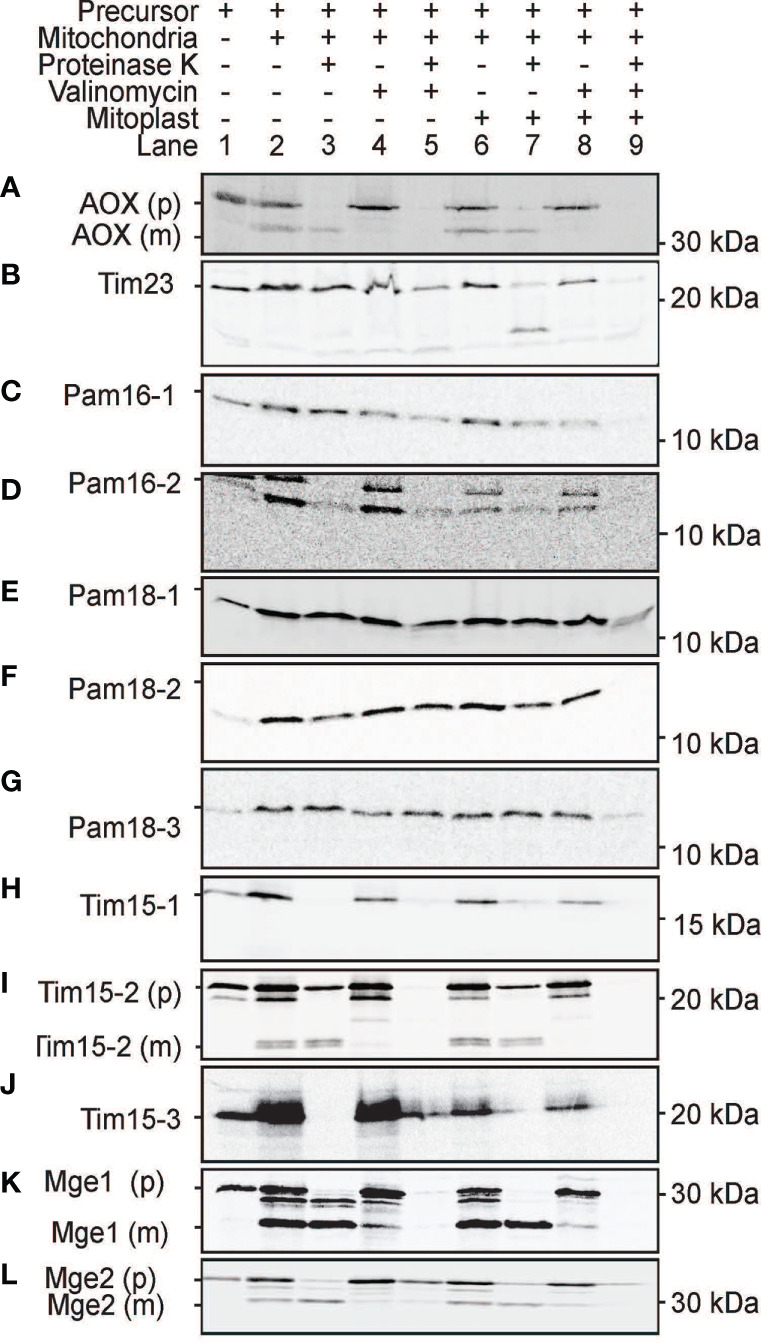
Protein import assays into isolated mitochondria. **(A)** Alternative Oxidase (AOX) was used as a control for successful import into the mitochondrial matrix while Tim23-2 **(B)** was used to test for the successful rupture of the outer membrane by osmotic shock following protein import. Pam16 **(C, D)**. Pam18 **(E–G)**. Tim15 **(H–J)**. Mge **(K, L)**. Lane 1 = precursor protein alone, lane 2 = precursor protein incubated with mitochondria under conditions that support import, lane 3 = as lane 2 with the addition of Proteinase K (PK) following import, lane 4 = same as lane 2 but with the addition of valinomycin prior to import, lane 5 = same as lane 4 but with the addition of PK following import, lane 6 = same as lane 2 but with outer membrane ruptured after import, lane 7 = same as lane 6 but with the addition of PK following import, lane 8 = same as lane 6 but with valinomycin prior to import, lane 9 = same as lane 8 but with PK treatment. (p) = precursor protein band. (i) = intermediate protein band. (m) = mature protein band. Position of protein size marker is shown on the right.

**Figure 9:**
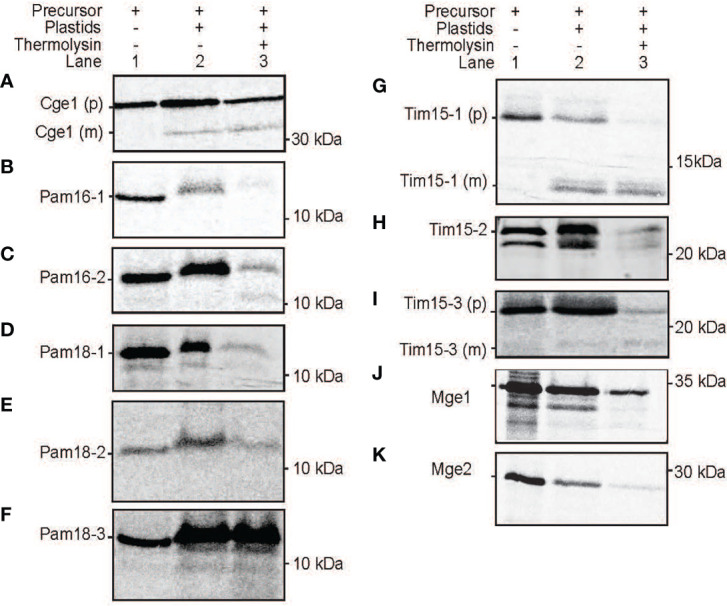
Protein import assays into isolated plastids. Import of radiolabelled proteins into isolated Arabidopsis plastids. Cge2 was used as a control for successful import **(A)**. Pam16 **(B, C)**, Pam 18 **(D-F)**, Tim15 **(G-I)**, Mge1 **(J, K)**. Lane 1 = precursor protein alone, lane 2 = precursor protein incubated with chloroplasts under conditions that support import, lane 3 = as lane 2 with the addition of thermolysin following import. (p) = precursor protein band. (m) = mature protein band. Position of protein size marker is shown on the right.

As the use of GFP fusion proteins can be error prone, (Duchêne et al., 2005; Sharma et al., 2018) we tested the targeting ability of PAM subunits without GFP tags. This was achieved by *in vitro* protein import assays using radiolabeled proteins imported into freshly isolated intact Arabidopsis mitochondria and plastids (**Figures 8** and **9**). For mitochondrial protein import assays, outer membranes were ruptured following protein uptake to confirm a proteins intra-mitochondrial localization, which is essential for proteins that do not contain a cleavable presequence (Murcha and Whelan, 2015). The Alternative Oxidase (AOX) and Tim23-2 proteins were used as controls to confirm protein uptake ability of the isolated mitochondria and to determine successful rupture of the outer membrane as previously described (Duncan et al., 2015). Import of the AOX precursor protein (AOX (p)) shows the generation of the mature protein (AOX (m)) upon successful import that is valinomycin sensitive (**Figure 8A**). Import of Tim23-2 reveals the generation of a ~14 kDa inner membrane PK protected fragment, confirming the successful import and rupture of the outer membrane (**Figure 8B**, lane 7). Import of Pam16 and Pam18 orthologues into isolated mitochondria showed the presence of a PK protected band in lane 3 and lane 7, confirming import through the inner membrane in a membrane potential dependent manner, with no processing observed (**Figures 8C–G**). Import of Tim15-1, and Tim15-3 did not suggest import into isolated mitochondria (**Figures 8H, J**), whilst Tim15-2 was imported and processed into a mature band of ~15 kDa (**Figure 8I**). Interestingly, a doublet band was visible suggesting an additional processing event may occur after presequence removal by MPP (**Figure 8I**). Import of Mge1 and Mge2 showed import and processing to mature proteins of ~25 and of 28 kDa respectively (**Figures 8K, L**). Both proteins show the presence of an intermediate processing product in lanes 2, 3 and 6, suggesting an additional processing event within the IMS (**Figures 8K, L**).

All PAM orthologues were tested for import into isolated Arabidopsis plastids. The plastid Cge1 was used as a control, showing import and processing to the mature protein of ~ 30 kDa size (**Figure 9A**). The mitochondrial Pam16-1 did not show import into isolated chloroplasts with no band present in lane 3 following thermolysin treatment, though Pam16-2 exhibited some weak binding and import (**Figures 9B,C**). Import of Pam18-1 and Pam18-2 similarly showed weak import with radiolabeled bands present in lane 3 whilst Pam18-3 showed strong binding and import (**Figures 9C–F**). Tim15-1 and Tim15-3 showed import and to mature products of ~10 kDa (**Figures 9G, I**) whilst Tim15-2 did not show import into isolated chloroplasts (**Figure 9H**). The dual-targeted Mge1 exhibited import into isolated chloroplasts, though no processing to a mature protein was observed (**Figure 9J**). whilst the mitochondrial Mge2 did not exhibit import into isolated chloroplasts (**Figure 9K**). Therefore, *in vitro* protein import assays show that Pam16 and Pam18 orthologues have the ability to be imported into isolated mitochondria whilst Pam18 orthologues were targeted to isolated plastids.

## Discussion

Pam16 and Pam18 are co-chaperones crucial for protein import into mitochondria. Plants contain multiple copies of Pam16 and Pam18 and whilst several studies have shown that Pam16 and Pam18 are targeted to mitochondria, none of these determined the localization of Pam18-1 (Tamadaddi et al., 2021). Furthermore, studies have shown that orthologues of PAM interacting partner proteins, Tim15 and Mge1, are localized to the mitochondria and plastids (Van Aken et al., 2009; Kluth et al., 2012; Langner et al., 2014). Therefore, we reexamined the targeting ability of these proteins utilizing multiple *in vitro* and *in vivo* approaches (**Table 1**). We found that Pam16-1 and Pam16-2 were targeted exclusively to the mitochondria using all methods tested such as transient biolistic transformation into cell suspensions, stable transgenic GFP-tagged lines, and *in vitro* protein import assays. We determined that Pam18 orthologues could be targeted to both mitochondria and plastids depending on the methodology used. For instance, Pam18-1 was exclusively targeted to the plastids in the biolistic transformation of cell suspensions, dual-targeted to plastids and mitochondria in the stable transgenic GFP lines using both the 35S promoter and native promoter and could be imported into isolated mitochondria and plastids. Pam18-2 was dual-targeted in transient biolistic transformations, exclusively targeted to mitochondria in stable transgenic GFP plants and could be imported into isolated mitochondria and plastids. Pam18-3 was found to be dual-targeted to mitochondria and chloroplasts, in both biolistic transformations and stable GFP lines but only localized to the mitochondria when using a native promoter (**Table 1**).

**Table 1:** Summary of results.

Protein name	Locus	Protein size.	GFP transient	GFP *in planta* 35S prom.	GFP *in planta* native prom.	M import	P import	Publications
Pam16-1	At3g59280	116 aa, 13 kDa	M	M	ND	Yes	No	(Huang et al., 2013) This study
Pam16-2	At5g61880	113 aa, 12 kDa	M	M	ND	Yes	Yes	(Huang et al., 2013) This study
Mge2	At4g26780	327 aa, 36 kDa	M	ND	ND	Yes	No	(Hu et al., 2012) This study
Tim15-2	At3g54826	223 aa, 25 kDa	M	ND	ND	Yes	No	(Kluth et al., 2012) This study
Pam18-1	At2g35795	112 aa, 12 kDa	P	D	D	No	No	This study
Pam18-2	At3g09700	112 aa, 12 kDa	D	M	M	No	Yes	(Tamadaddi et al., 2021) This study
Pam18-3	At5g03030	112 aa, 12 kDa	D	D	M	No	Yes	(Tamadaddi et al., 2021) This study
Mge1	At5g55200	302 aa, 33 kDa	D	ND	ND	Yes	Yes	(Van Aken et al., 2009; Langner et al., 2014; Sharma et al., 2018)
Tim15-1	At1g68730	170 aa, 19 kDa	P	ND	ND	No	Yes	(Kluth et al., 2012) This study
Tim15-3	At5g27280	212 aa, 23 kDa	P	ND	ND	No	Yes	(Kluth et al., 2012) This study
Cge1	At5g17710	279 aa, 31 kDa	P	ND	ND	No	Yes	(de Luna-Valdez et al., 2019) This study
Cge2	AT1g36390	326 aa, 36 kDa	P	ND	ND	ND	ND	(de Luna-Valdez et al., 2019) This study

aa, amino acids; ND, Not determined in this study; M, Mitochondria; P, Plastid– D, Dual-targeted to both organelles; Prom, Promoter.

Localization for Pam16, Pam18, Tim15, Mge1 and Cge orthologues as determined by GFP-tagged biolistic transformation, GFP-tagged transgenic lines with either a 35S or native promoter and *in vitro* import assays into isolated organelles.

Whilst Pam18-2 and Pam18-3 localization has been investigated previously (Tamadaddi et al., 2021), localization was only tested in the root cells of stable GFP fusion lines suggesting that any potential plastid localization may not have been observed. Here, Pam18’s unexpected plastid localization was dependent on protein abundance and cell specific factors, highlighting the need for multiple and comprehensive experimental approaches. This has been previously shown with other dual-targeted proteins. The targeting of Mge1 was tested using various methods such as GFP-fusions delivered *via* biolistic transformation, protoplasts, agrobacterium transformation, and Mge1 showed both mitochondrial or dual-targeting ability (Sharma et al., 2018) as we confirmed here. Plastids are known to acquire specialized and distinct functions for photosynthesis, metabolism and storage and thus their proteomes differ in various cell types (Wise, 2007). Furthermore, recent studies have identified specialized stress sensing plastids known as sensory plastids (Dopp et al., 2021) and that the dual-targeted protein MutS Homolog1 (MSH1) involved in organelle genome functions (Xu et al., 2011) has been shown to reside exclusively in epidermal sensory plastids (Beltrán et al., 2018). This work also raises the possibility that Pam18 also plays a specialized role in plastids. Plastids, like mitochondria, evolved from an independent endosymbiotic event and similarly lost the majority of its genome, thus requiring import of the majority of its proteome to carry out its essential functions. Whilst each organelle possesses mostly unique and evolutionary distinct protein import machinery, the general mechanisms are strikingly similar (Flores-Pérez and Jarvis, 2013; Langner et al., 2014; Ghifari et al., 2018; Nakai, 2018). Both mitochondria and plastids utilize targeting peptides to deliver the cytosolic proteins to the organelle, which is cleaved upon import. Several of the proteases and aminopeptidases involved in the sequential degradation of the targeting signal have been shown to be dual-targeted to mitochondria and plastid. The Presequence Peptidase (PreP), Organellar oligopeptidase (OOP) and the M1 and M17 family of amino peptidase are all dual-targeted and play vital roles in plant homeostasis (Moberg et al., 2003; Kmiec et al., 2013; Kmiec et al., 2018; Ghifari et al., 2022). Whilst not a dual-targeted protein, the plant mitochondrial outer membrane receptor 64 (OM64) is paralogous to the plastid outer envelope receptor TOC64 (Lister et al., 2007). Both receptors function by binding to the cytosolic Hsp70 and Hsp90 *via* their TPR domains to initiate protein translocation across the outer membrane/envelope (Panigrahi et al., 2014; Nickel et al., 2019).

Both organelles utilize Hsp70 to translocate proteins in an ATP-dependent manner alongside co-chaperones and nucleotide exchange factors (Schilke et al., 2017; Clerico et al., 2019). The versatility of Hsp70 in such diverse mechanisms such as protein import, preventing protein aggregation, and folding of nascent polypeptide chains lies in its interaction with co-chaperones (Kampinga and Craig, 2010; Schilke et al., 2017). In plastids, cpHsp70s have been shown to interact with protein import components such as the inner envelope Tic40 and Tic110 (Nakai, 2018), both of which have shown to also associate with Cge1 (de Luna-Valdez et al., 2019). However, to date, no dedicated J-domain co-chaperones has been identified to interact with cpHsp70 or the plastid protein import systems (Flores-Pérez and Jarvis, 2013; Kikuchi et al., 2013). In Arabidopsis Mge1 is dual-targeted to mitochondria and plastids, and Mge2 is exclusively mitochondrial (Van Aken et al., 2009) (Hu et al., 2012). Whilst the specific role of Mge1 has yet to be determined the presence of two Mge homologs is conserved throughout plants suggesting diverged roles (Hu et al., 2012). Both Mge1 and 2 are highly stress responsive proteins and it has been suggested that Mge1 has a role in general protein import whilst the thermotolerant Mge2 plays a specific role when plants are exposed to high temperatures (Van Aken et al., 2009; Hu et al., 2012; Maziak et al., 2021). Though it remains to be seen what role Mge1 plays in the plastid.

Orthologues of Tim15 (HEP/ZR) another Hsp70 PAM complex co-chaperone has also been observed to be targeted to the mitochondria and plastid. Tim15-1 was shown to physically interact with mtHSP70 and was able to complement a yeast deletion strain (Kluth et al., 2012) with a likely role in preventing mtHSP70 aggregation (Nyakundi et al., 2016). Tim15-1 and Tim15-3 both have been confirmed as plastid proteins (Kluth et al., 2012) and similarly found to interact with cpHsp70 (Willmund et al., 2008). Plastid Tim15 was observed to be able to stimulate the ATPase activity of cpHSP70 required for protein import in moss (Liu et al., 2014) supporting this co-chaperones role in chloroplast protein import.

Both mtHsp70 and cpHsp70 show little divergence on a protein sequence level with their functions and specificity being regulated by their interactions with co-chaperones (Miernyk, 2001). The ability of Pam18 orthologues to be targeted to the plastid raises the possibility that the Pam18’s may also function as cpHSP70 co-chaperones alongside Mge and Tim15, to function in a developmental stress responsive and/or tissue specific manner. Pam18 transcript abundance profiles show that the dual-targeted Pam18-3 does not show high abundance in seed, a developmental stage where protein import components including Pam16’s are most abundant (Narsai et al., 2011; Chen et al., 2013). Furthermore, Pam18 homologues exhibit differential stress responses with regards to heat and drought (Chen et al., 2013) suggesting sub-functionalization. Recently, it has been shown that Pam18 has additional functions with regards to interacting with the respiratory chain in the mitochondrial inner membrane (Wiedemann et al., 2007; Priesnitz et al., 2022) promoting the assembly of Complex IV (Priesnitz et al., 2022). More interestingly, the function of mitochondrial Pam16 and Pam18 has been totally replaced by an unrelated protein Pam27 in trypanosomes (von Känel et al., 2020) which highlight the divergence and dynamic nature of protein import components and mechanisms across eukaryotes.

## Data availability statement

The datasets presented in this study can be found in online repositories. The names of the repository/repositories and accession number(s) can be found in the article/**Supplementary Material**.

## Author contributions

MG-H and MWM; research design; MG-H, AW and MWM; conducting experiments and data analysis; MG-H, AW and MWM preparation of the manuscript. All authors contributed to the article and approved the submitted version.

## Funding

MWM is supported by an Australian Research Council Discovery Project (DP200101922). MG-H is supported by a University Postgraduate Award at the University of Western Australia. AW was supported by a Rowe Scientific Foundation Vacation Award.

## Conflict of interest

The authors declare that the research was conducted in the absence of any commercial or financial relationships that could be construed as a potential conflict of interest.

## Publisher’s note

All claims expressed in this article are solely those of the authors and do not necessarily represent those of their affiliated organizations, or those of the publisher, the editors and the reviewers. Any product that may be evaluated in this article, or claim that may be made by its manufacturer, is not guaranteed or endorsed by the publisher.

## Supplementary material

Supplementary Figure 1 PAM complex subunits are conserved across species. **(A, B)** Phylogenetic analysis of Pam16, Pam18 and Tim15 orthologues from *Saccharomyces cerevisiae* (Sc), *Arabidopsis thaliana* (At), *oryza sativa* (Os), *Caenorhabditis elegans* (Ce), *Zea mays* (Zm), *Homo sapiens* (Hm), *cyanobacteria* (Cn), and *Selaginella moellendorffii* (Sm) PAM orthlogues with known localisation are highlighted in yellow (mitochondria) or green (chloroplast) respectively. **(C)** Protein features of PAM subunits; inner membrane space domain (IMS) domain (pink), transmembrane region (yellow), J-domain or J-like domain (purple, IPR001623) and a histidine-proline-aspartic acid (HPD) motif (red). The zinc ribbon superfamily domain present in Tim15 (green) and the zinc finger C-C motif (orange). **(D)** Predicted tertiary structures of the Arabidopsis Pam16, Pam18 and Tim15 orthologues overlaid over the experimentally determined structure of its putative yeast orthologue.

Supplementary Figure 2 Conserved protein domains and motifs in Pam16, Pam18, Tim15, Mge1 and Cge orthologues from yeast and Arabidopsis. Domain mapping was done using InterPro (https://www.ebi.ac.uk/interpro/) and ScanProsite (https://prosite.expasy.org/scanprosite/). All Pam16s contain an intermembrane space domain (IMS domain, pink), and a transmembrane region (yellow), and a J-like domain (blue). The Pam18s additionally contain a J-domain (purple) instead of J-like domain distinguished by the presences of the HPD motif (red). The Tim15s all contain the zinc ribbon superfamily domain (green) with a C–C motif (orange). The Mge1 and Cge orthologues all contain a similar GrpE superfamily domain (yellow).

Supplementary Figure 3 Bright field images corresponding to **Figure 4**. Scale bar indicates 20 mM.

Supplementary Figure 4 Bright field images corresponding to **Figures 5** and **6**. Scale bar indicates 20 mM.
