# Supplementary figures and images for: Presequence translocase-associated motor subunits of the mitochondrial protein import apparatus are dual-targeted to mitochondria and plastids
Source: Front Plant Sci. 2022 Nov 11;13:981552. doi: 10.3389/fpls.2022.981552 (PMC9695410; doi:10.3389/fpls.2022.981552)

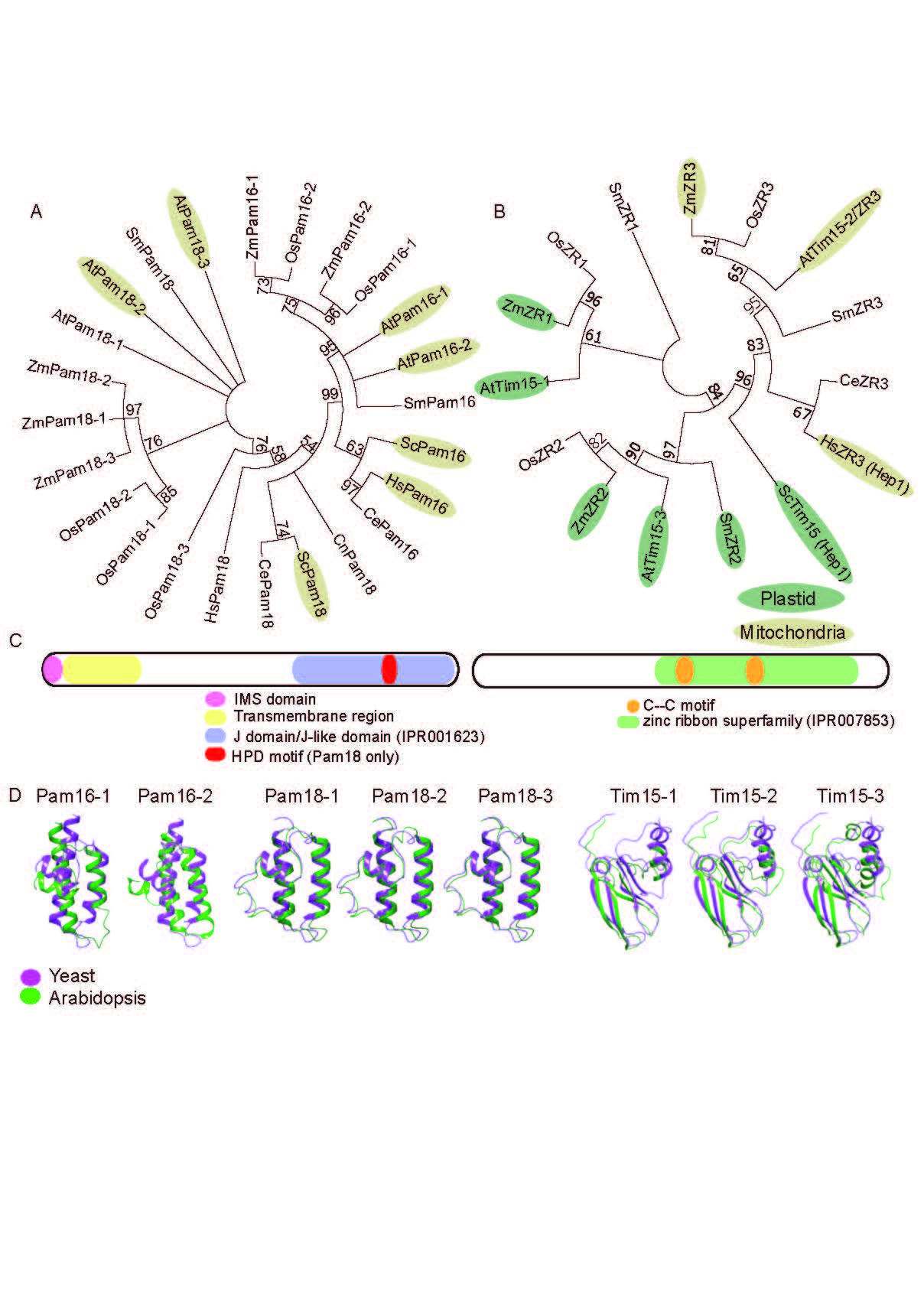

Supplement: Supplementary Figure 1 — PAM complex subunits are conserved across species. (A, B) Phylogenetic analysis of Pam16, Pam18 and Tim15 orthologues from Saccharomyces cerevisiae (Sc), Arabidopsis thaliana (At), oryza sativa (Os), Caenorhabditis elegans (Ce), Zea mays (Zm), Homo sapiens (Hm), cyanobacteria (Cn), and Selaginella moellendorffii (Sm) PAM orthlogues with known localisation are highlighted in yellow (mitochondria) or green (chloroplast) respectively. (C) Protein features of PAM subunits; inner membrane space domain (IMS) domain (pink), transmembrane region (yellow), J-domain or J-like domain (purple, IPR001623) and a histidine-proline-aspartic acid (HPD) motif (red). The zinc ribbon superfamily domain present in Tim15 (green) and the zinc finger C-C motif (orange). (D) Predicted tertiary structures of the Arabidopsis Pam16, Pam18 and Tim15 orthologues overlaid over the experimentally determined structure of its putative yeast orthologue. [file Image_1.jpeg]

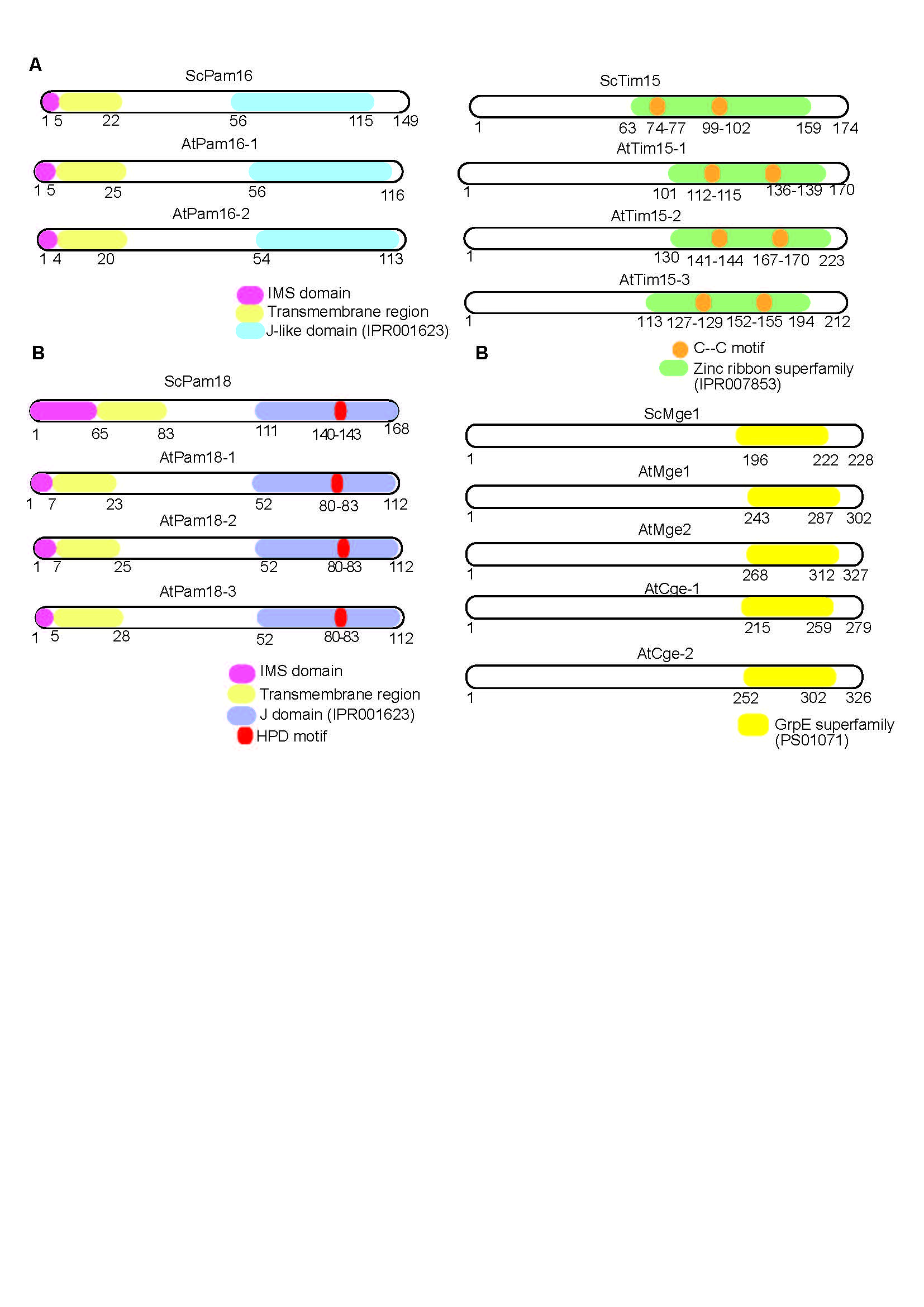

Supplement: Supplementary Figure 2 — Conserved protein domains and motifs in Pam16, Pam18, Tim15, Mge1 and Cge orthologues from yeast and Arabidopsis. Domain mapping was done using InterPro (https://www.ebi.ac.uk/interpro/) and ScanProsite (https://prosite.expasy.org/scanprosite/). All Pam16s contain an intermembrane space domain (IMS domain, pink), and a transmembrane region (yellow), and a J-like domain (blue). The Pam18s additionally contain a J-domain (purple) instead of J-like domain distinguished by the presences of the HPD motif (red). The Tim15s all contain the zinc ribbon superfamily domain (green) with a C–C motif (orange). The Mge1 and Cge orthologues all contain a similar GrpE superfamily domain (yellow). [file Image_2.jpeg]

Root

Epidermis

Guard cell

**A**

Pam16-1 At3G59280

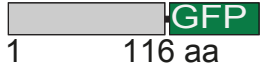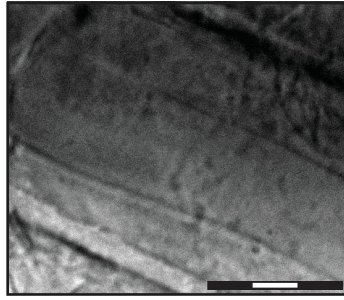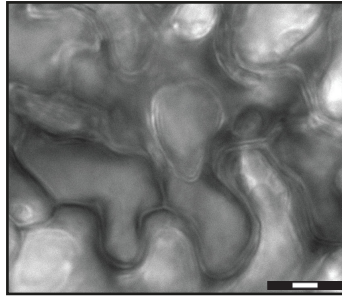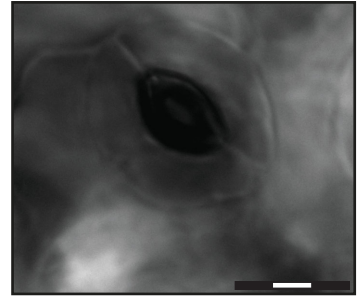

**B**

Pam16-2 At5G61880

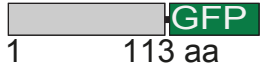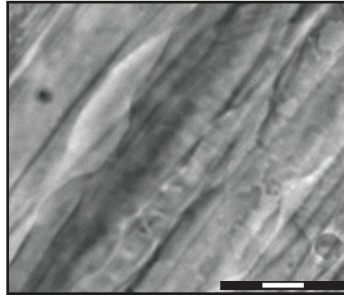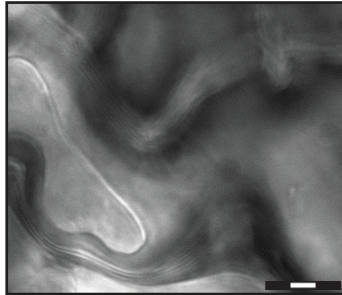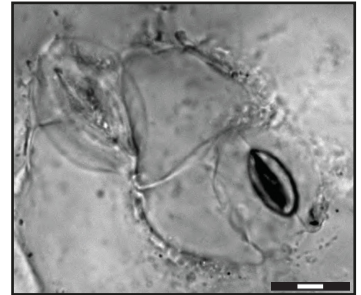

**C**

Pam18-1 At2G35795

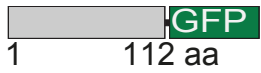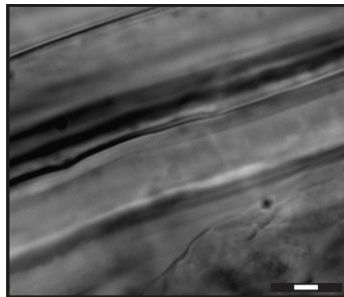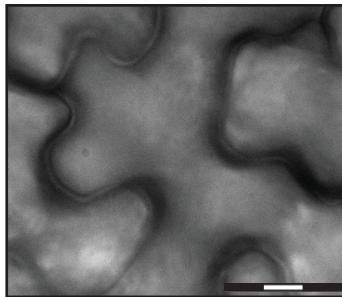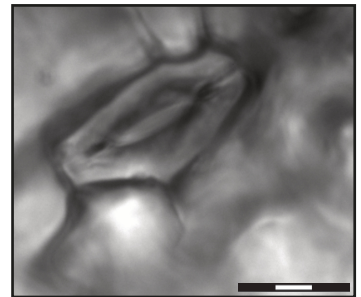

**D**

Pam18-2 At3G09700

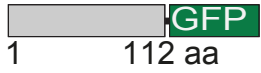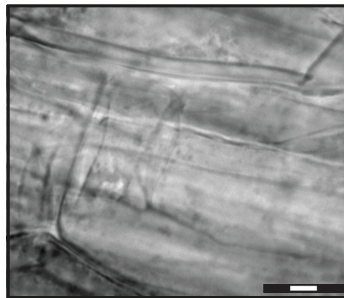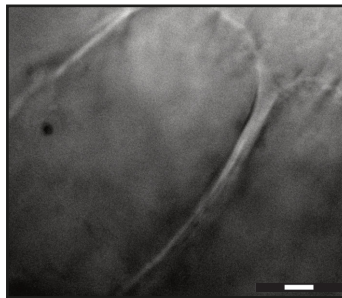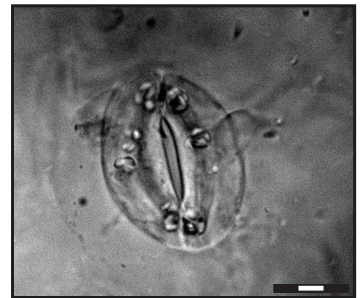

**E**

Pam18-3 At5G03030

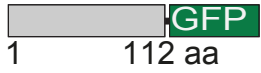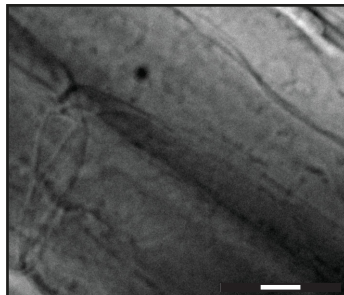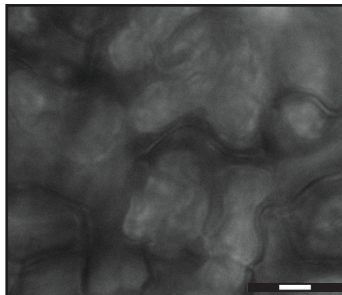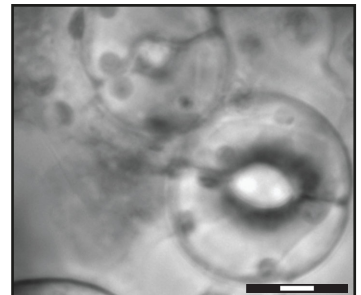

**F**

Col-0

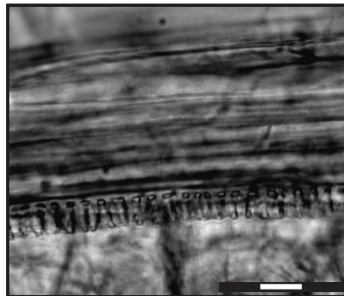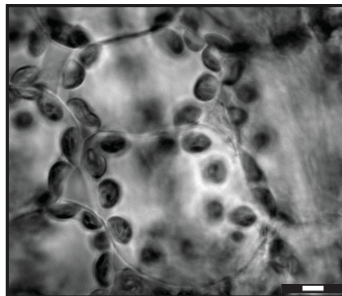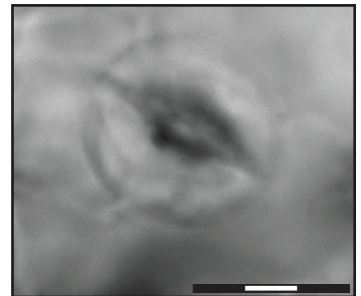

Supplement: Supplementary Figure 3 — Bright field images corresponding to Figure 4. Scale bar indicates 20 mM. [file Image_3.pdf]

Epidermis

Guard cell

Root cell

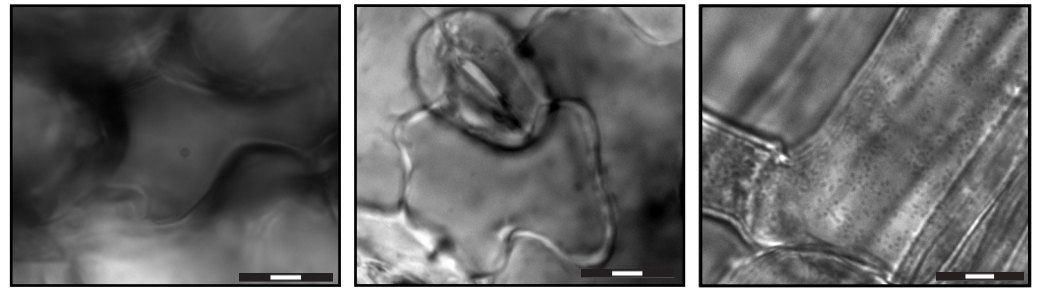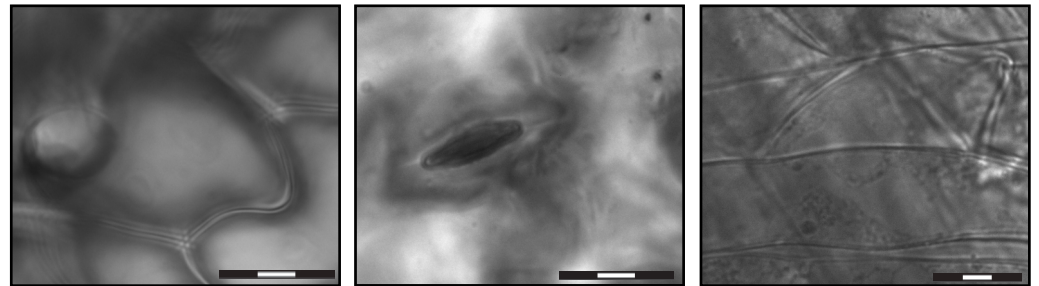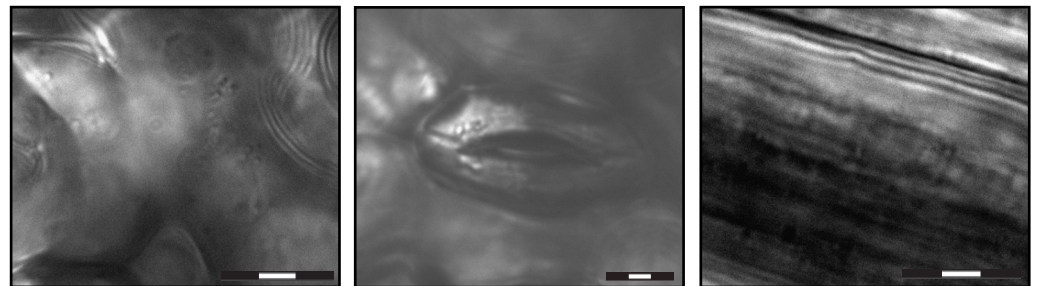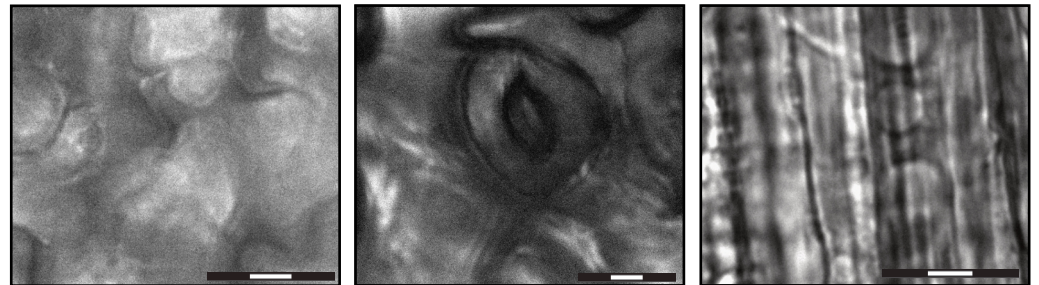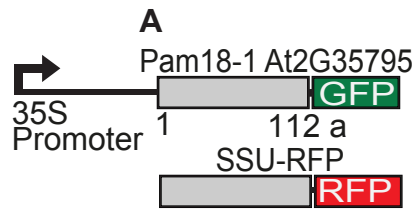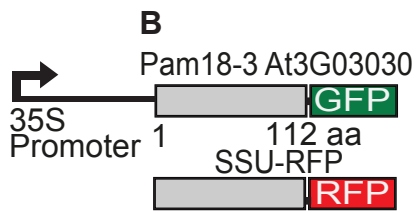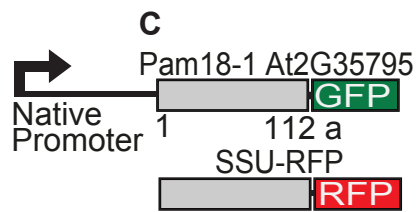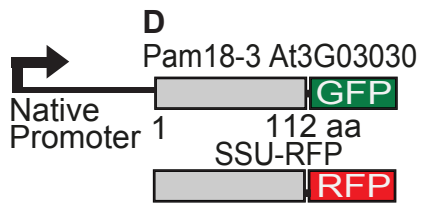

Supplement: Supplementary Figure 4 — Bright field images corresponding to Figures 5 and 6. Scale bar indicates 20 mM. [file Image_4.pdf]
